# Glucopyranosyl Lipid A Adjuvant Significantly Enhances HIV Specific T and B Cell Responses Elicited by a DNA-MVA-Protein Vaccine Regimen

**DOI:** 10.1371/journal.pone.0084707

**Published:** 2014-01-23

**Authors:** Paul F. McKay, Alethea V. Cope, Jamie F. S. Mann, Sarah Joseph, Mariano Esteban, Roger Tatoud, Darrick Carter, Steven G. Reed, Jonathan Weber, Robin J. Shattock

**Affiliations:** 1 Imperial College London, Department of Infectious Diseases, Division of Medicine, Norfolk Place, London, United Kingdom; 2 MRC Clinical Trials Unit, London, United Kingdom; 3 Centro Nacional de Biotecnología, CSIC, Department of Molecular and Cellular Biology, Campus Universidad Autónoma, Madrid, Spain; 4 Infectious Disease Research Institute, Seattle, Washington, United States of America; Commissariat a l'Energie Atomique(cea), France

## Abstract

Using a unique vaccine antigen matched and single HIV Clade C approach we have assessed the immunogenicity of a DNA-poxvirus-protein strategy in mice and rabbits, administering MVA and protein immunizations either sequentially or simultaneously and in the presence of a novel TLR4 adjuvant, GLA-AF. Mice were vaccinated with combinations of HIV *env*/*gag*-*pol*-*nef* plasmid DNA followed by MVA-C (HIV *env*/*gag*-*pol*-*nef*) with HIV CN54gp140 protein (+/−GLA-AF adjuvant) and either co-administered in different muscles of the same animal with MVA-C or given sequentially at 3-week intervals. The DNA prime established a population of B cells that were able to mount a statistically significant anamnestic response to the boost vaccines. The greatest antigen-specific antibody response was observed in animals that received all vaccine components. Moreover, a high proportion of the total mucosal IgG (20 – 50%) present in the vaginal vault of these vaccinated animals was vaccine antigen-specific. The potent elicitation of antigen-specific immune responses to this vaccine modality was also confirmed in rabbits. Importantly, co-administration of MVA-C with the GLA-AF adjuvanted HIV CN54gp140 protein significantly augmented the antigen-specific T cell responses to the Gag antigen, a transgene product expressed by the MVA-C vector in a separate quadriceps muscle. We have demonstrated that co-administration of MVA and GLA-AF adjuvanted HIV CN54gp140 protein was equally effective in the generation of humoral responses as a sequential vaccination modality thus shortening and simplifying the immunization schedule. In addition, a significant further benefit of the condensed vaccination regime was that T cell responses to proteins expressed by the MVA-C were potently enhanced, an effect that was likely due to enhanced immunostimulation in the presence of systemic GLA-AF.

## Introduction

Designing an immunization strategy to induce potent cellular immunity together with systemic and mucosal humoral responses may prove critical in the development of an effective vaccine against HIV-1 infection. Interest in multi-component or heterologous vaccine regimes has increased in recent years. This is due in part to the RV144 Thai clinical trial of a prime/boost regime using recombinant canary poxvirus (ALVAC-HIV) and two gp120 proteins (AIDSVAX B and E), that demonstrated modest vaccine efficacy (31%) against HIV-1 acquisition, and is further supported by a larger body of evidence from non-human primate studies that demonstrate significant immunological benefit of multi-component vaccination strategies [1]–[4]. Heterologous vaccination is based on two core concepts. First, that the use of different vaccine modalities (plasmids, viral vectors, and/or proteins) reduces the potential elicitation of anti-vector immunity that would diminish the potency and hence immunity-boosting potential of repeat vaccinations [5], [6]. Second, that the use of different vaccine components and/or their order of administration can be used to tailor resulting immunity, both quantitatively and qualitatively [7], [8]. Although there is clear evidence to support the former concept [9], [10], our understanding of how to tailor vaccine-elicited immunity through the heterologous use of different vaccine components is still very much in its infancy [11]–[15].

In this context, plasmid DNA is known to effectively prime CD4 helper, CD8 cytotoxic T cells and humoral immune responses, readily detectable after recombinant vector or protein boost vaccinations [16], [17]. In contrast, poxviral vectors such as MVA, although relatively poor at priming adaptive immunity, have been successfully used to boost and expand pre-existing CD4 and CD8 cellular immunity [18], [19]. Recombinant protein on the other hand, is thought to be optimal for stimulating potent antigen-specific B cell responses, particularly when administered with an adjuvant [16], [20]. Previous studies by us and others have demonstrated potent enhancement of both cellular and humoral immune responses using TLR4 agonist adjuvants [21]–[23]. While each of these individual components have the ability to elicit immune responses, none are thought to be sufficient by themselves to generate effective levels of both cellular and humoral immunity.

An additional consideration for the heterologous use of different vaccine components is to increase the breadth of induced immune responses. In this respect, responses focused too narrowly on a few highly immunodominant epitopes can lead rapidly to immune escape, particularly in the case of HIV-1 with its very high capacity for mutation [24], [25]. Furthermore, the broad genetic diversity of HIV-1 even within individual clades provides a significant challenge for immunogen design where induced neutralizing antibody responses to individual recombinant proteins are often isolate specific [26]. Plasmid DNA is thought to effectively prime broad cellular responses [27], [28] that can be effectively boosted by viral vectors including MVA. Moreover, humoral responses to recombinant proteins, primed by DNA and/or viral vectors are larger and more diverse with respect to breadth of neutralization than those elicited by protein alone [29]–[31]. Such differences in terms of magnitude and breadth can be further influenced by the provision of additional immunostimulation through the incorporation of adjuvant moieties [32]–[36].

In this study we assess the immunogenicity in mice of plasmid DNA, MVA poxvirus and recombinant protein vaccine combinations using matched HIV-1 Clade C Env gp140 and Gag-Pol-Nef fusion antigens to determine the optimal vaccine regimen for testing in a human clinical trial. We used an aqueous glucopyranosyl lipid A (GLA-AF) adjuvant formulation to augment the HIV CN54gp140 protein component of our vaccine. We determined the relative immunogenicity of the individual vaccine components when administered sequentially. Here animals were primed using plasmid DNA to enhance immune breadth prior to booster vaccinations with MVA and recombinant protein. However, although sequential use of the individual components offered potential for enhanced immune responsiveness, it also significantly extended the duration of the vaccination regime, potentially impacting on the feasibility of its implementation in future human clinical trials. Therefore we also looked for evidence for cross-talk between the different vaccine components and assessed the potential positive or negative consequences of shortening the vaccine regimen by co-administration rather than sequential application of the MVA-C, CN54gp140 protein with or without GLA-AF adjuvant.

## Materials and Methods

### Ethics Statement

The animal studies were approved by the Ethical Review Board of Imperial College and work was performed in compliance with project and personal animal experimentation licences granted by the UK government in accordance with the Animals in Scientific Procedures Act (1986).

### Recombinant trimeric HIV-1 gp140, plasmid and viral vectors, GLA-AF

A clade C HIV-1 envelope clone, designated p97CN54, was obtained from an HIV infected Chinese patient [37], [38], codon optimized by GeneArt (Invitrogen, UK) was made available by Prof H. Wolf and Prof R. Wagner (University of Regensburg, Germany). Trimeric gp140 clade C envelope (gp120 plus the external domain (ED) of gp41), designated CN54gp140, was produced as a recombinant product in CHO cells (S. Jeffs - personal communication), and the protein manufactured to GMP specification by Polymun Scientific (Vienna, Austria). The identity of the product was confirmed by mass spectrometric analysis of tryptic fragments by the Medical Biomics Centre at St. George's, University of London. The trimeric product was stable, and has been extensively tested to validate stability even when kept at room temperature (D. Katinger - personal communication) and has previously been reported to be immunogenic [39], [40]. Two plasmid DNA vectors were used in these studies. One contained the CN54 *Env* transgene and the other a *Gag-Pol-Nef* genetic fusion construct. Both plasmids utilized CMV enhancer/promoter with a human T-cell leukemia type 1 regulatory element to drive transgene expression [41]. The MVA pox vector was created by Prof. Mariano Esteban (CSIC, Spain) and expresses the CN54gp120 Env and the Gag-Pol-Nef polyprotein from two back-to-back synthetic Early/Late transcriptional promoters [42], [43]. The micellar formulation of GLA has been denoted previously as IDRI-AQ001 and is more generally denoted as GLA-AF [44]. The biological and physicochemical characterization of GLA has been published previously [45].

### Mice, immunization and sampling

Female BALB/c mice (Harlan, UK), 6–8 weeks old, were placed into groups (n = 10) and housed in a fully acclimatized room. All animals were handled and procedures performed in accordance with the UK Home Office Animals (Scientific Procedures) Act 1986 in accordance with an internal ethics board and a UK government approved project and personal licence awarded to the corresponding author. Food and water were supplied *ad libitum*. Mice were initially immunized at 3 week intervals with three intramuscular 100 µg (50 µg each leg) plasmid DNA vaccinations (two plasmids, one containing an HIV CN54gp140 *Env* transgene injected into the left hind quadriceps muscle in a volume of 50 µl and the other an HIV ZM96 *Gag*, *Pol and Nef* fusion transgene injected into the right hind quadriceps muscle also in a 50 µl volume, both constructs together being termed DNA-C for clarity). This was followed by a 6 week rest period where the animals did not receive any vaccinations. Mice then received either two or four further vaccinations at 3 week intervals with various combinations of IM delivered recombinant poxvirus MVA *env*-*gag*-*pol*-*nef* (MVA-C) at 10^7^ PFU in 50 µl and/or recombinant gp140 with and without the TLR4 agonist GLA-AF (IDRI, Seattle, USA). CN54gp140 was administered at 10 µg per 50 µl dose and if GLA-AF was used it was co-administered with the CN54gp140 protein at 20 µg per 50 µl dose. Tail bleeds were collected before the start of the protocol and one day prior to each vaccination without anti-coagulant and centrifuged in a Heraeus Biofuge *pico* (Fisher, UK) at 1000 g for 10 min. The serum was harvested and transferred into fresh 0.5 ml micro-centrifuge tubes (Starlabs, UK), and stored at −20°C until antigen-specific antibody concentrations were determined by indirect quantitative ELISA. Vaginal lavage was carried out immediately before the tail bleeds using three 25 µl washes/mouse with sterile PBS that were subsequently pooled. Lavage samples were incubated for 30 min with 4 µl of 25× stock solution protease inhibitor (Roche Diagnostics, Germany) before centrifuging at 1000 g for 10 min. The fluid supernatant from these treated samples was then transferred into a fresh 0.5 ml micro-centrifuge tube, and stored at −20°C until antigen-specific and the total non-specific antibody concentrations were determined by indirect quantitative ELISA.

### Rabbits, immunization and sampling

Male and female New Zealand White rabbits (HsdIf:NZW; Harlan, UK), 10–11 weeks old and with weights ranging from 2.0–2.5 kg for males and 2.0–2.4 kg for females, were placed into groups (n = 14; 7 males and 7 females per group) and housed in a fully acclimatized room. All animals were handled and procedures performed in accordance with the UK Home Office Animals (Scientific Procedures) Act 1986 in accordance with an internal ethics board and a UK government approved project and personal licence awarded to the corresponding author. Food and water were supplied *ad libitum*. Rabbits were immunized at 3 week intervals with three intramuscular 8 mg (4 mg each leg) plasmid DNA vaccinations (two plasmids, one containing an HIV CN54gp140 *Env* transgene injected into the left hind quadriceps muscle in a volume of 1 ml and the other an HIV ZM96 *Gag*, *Pol and Nef* fusion transgene injected into the right hind quadriceps muscle also in a 1 ml volume). This was followed by a 6 week rest period where the animals did not receive any vaccinations. The animals then received either two or four further vaccinations at 3 week intervals with various IM administered combinations of a recombinant poxvirus (MVA *env*-*gag*-*pol*-*nef* (MVA-C) at 1.3×10^8^ PFU in 500 µl and/or recombinant gp140 with and without the TLR4 agonist GLA-AF. CN54gp140 was administered at 100 µg per 400 µl dose and if GLA-AF was used it was co-administered with the CN54gp140 protein at 5 µg per 400 µl dose. Ear bleeds (auricular artery) were collected before the start of the protocol and one day prior to each vaccination without anti-coagulant and centrifuged in a Heraeus Biofuge *pico* (Fisher, UK) at 1000 g for 10 min. The serum was harvested and transferred into fresh 0.5 ml micro-centrifuge tubes (Starlabs, UK), and stored at −20°C until antigen-specific antibody levels were determined by indirect quantitative ELISA. Vaginal vestibular sampling was carried out at autopsy of the female animals. Briefly, two Weck-Cel (Medtronic, UK) spears were used per female rabbit, one being placed on the mucosal surface of the vaginal and another on the mucosal surface of the vestibule for 2 min to soak up any secretion present. The spears were then removed and placed into the top chamber of a Spin-X tube that contained 300 µl of a high salt extraction buffer (100 ml PBS, 1.5 g NaCl, 1 ml 100× Protease Inhibitors, 20 ul 10% NaN3). Each spear head was cut from the stalk and the handle discarded and the tubes were then frozen at ≤55°C until required for further processing which consisted of centrifugation of the thawed tube for 30 min at 14,000 rpm through the Spin-X filter and then immediate analysis of the mucosally sourced sample by indirect quantitative ELISA.

### Anti-CN54gp140 antibody quantitative ELISA

Serum and mucosal antigen-specific gp140 binding antibodies against recombinant CN54gp140 were measured using a standardized ELISA. Maxisorp high binding 96-well plates were coated with 100 µl recombinant CN54 gp140 at 5 µg/ml in PBS for overnight at 4°C. The standard immunoglobulins were captured with a combination of anti-murine lambda and kappa light chain specific antibodies (Serotec, UK). These capture antibodies were coated onto the maxisorp plates overnight at 4°C (100 µl of a 1∶3200 dilution; Serotec). Coated plates were washed three times in PBS-T before blocking with 200 µl PBS-T containing 1% bovine serum albumin for 1 hour at 37°C. After further washing, sera diluted 1/100 or mucosal wash samples diluted 1/10 were added to the antigen coated wells and a standard titration of immunoglobulin standards added to the kappa/lambda capture antibody coated wells at 50 µl/well and incubated for 1 hour at 37°C. Plates were washed four times before the addition of 100 µl of a 1/4,000 dilution of goat anti-mouse Ig-HRP (various isotypes - matched to the standard immunoglobulin isotype; Southern Biotech) secondary antibody and incubated for 1 hour at 37°C. The plates were washed four times and developed with 50 µl/well of KPL SureBlue TMB substrate (Insight Biotechnology, UK). The IgA isotype, that was biotin labeled, required a further Streptavidin-HRP (R&D systems) amplification step prior to TMB development. The reaction was stopped after 15 min by adding 50 µl/well 1 M H_2_SO_4_, and the absorbance read at 450 nm on a KC4 spectrophotometer.

### IFN-gamma T Cell ELISpot

Spleens were harvested from each animal and processed as described previously [23]. Antigen-reactive splenocyte T cells were enumerated using a standardized IFN-gamma T cell ELISpot (Shattock lab SOP). Briefly, IFN-gamma capture antibody was coated overnight on ethanol activated HTS multiscreen (Millipore) plates at 4°C, then washed with sterile PBS. 50 µl of splenocytes (5×10^6^ cells/ml) were added to each well together with various Env or Gag peptide pools (15-mers overlapping by 11aa; each peptide used at a final concentration of 5 µg/ml; Env Pool 1 = 78 peptides, Env Pool 2 = 78 peptides, Gag Pool 1 = 61 peptides, Gag Pool 2 = 60 peptides) or positive and negative controls of ConA (5 µg/ml) or medium alone respectively (50 µl) and incubated for 16–18 hours before further washing and development of the spots using a streptavidin labelled IFN-gamma sandwich detection antibody and streptavidin alkaline phosphatase. Colour was developed with the BCIP/NBT substrate solution. ELISpot plates were read using an AID ELISpot reader ELR03 (Autoimmun Diagnostika GmbH, Ger). ELISpot well counts were defined as positive if they were more than twice the standard deviation of the background unstimulated cells and were above 50 spot forming units (SFU) after subtraction of the background.

### Intracellular Cytokine Staining and Analysis

Splenocytes were cultured at 37°C in a 5% CO2 environment for 6 hours in the presence of RPMI medium alone (unstimulated control) or Env and Gag peptide pools (specific responses – each peptide present at a concentration of 5 µg/ml with pools being the same as described for the ELISpot assays and total DMSO concentration was less than 2% in all final peptide pool stimulations) or 100 ng of phorbol myristate acetate (PMA) per ml plus 1 µg of ionomycin per ml (positive control). All cultures contained brefeldin A (GolgiPlug; BD Pharmingen) at 10 µg/ml to disrupt Golgi apparatus transport, thereby causing intracellular cytokine accumulation and the co-stimulatory antibodies anti-mouse CD28 and CD49d and were in a total volume of 100 µl. The cultured stimulated cells were washed then stained with live/dead fixable violet (Invitrogen, UK), washed again in PBS-2% FCS then incubated with mAbs specific for cell surface molecules (CD3-V500, clone 500A2; CD4-APC-Cy7, clone GK1.5 and CD8-PerCp Cy5.5, clone 53–6.7; all BD Biosciences, UK) prior to permeabilization and fixation where briefly, the cells were washed once with PBS-2% FCS and then permeabilized with Cell Fix/Perm solution (BD Pharmingen) in accordance with the permeabilization protocol. Cells were washed twice with 2.5 volumes of 1× Perm/Wash buffer and then stained with 1 µg of each anticytokine mAb per 10^6^ cells (IFNγ-Pe-Cy7, clone XMG1.2; TNFα-FITC, clone MP6-XT22 and IL-2-PE, clone JES6-5H4; all BD Biosciences, UK). Anticytokine mAb titers for optimal staining were determined in preliminary experiments. Cells were washed twice with 2 ml of 1× Perm/Wash buffer and once with PBS and then fixed in 1.5% formaldehyde-PBS. Samples were analyzed on a FACS CantoII instrument with FACS Diva software. Total single cell lymphocytes were gated using forward and side scatter and a ‘singlets’ gate prior to identification of any dead cells that had taken up the live/dead stain and 4×10^4^ gated and live CD3 positive T cells were collected. Positive cytokine responses were determined by using fluorescence minus one (FMO) to assess non-specific antibody staining and the use of an unstimulated control for each individual splenocyte sample to determine any background activation status giving a positive cytokine response. Gates were set using the FMO as the absolute guide and any background cytokine response in the unstimulated cells were subtracted from the specific peptide pool generated response. Data analysis was performed with FlowJo (Treestar Inc., OR, USA) and the analysis and presentation of distributions was performed using SPICE version 5.22, downloaded from http://exon.niaid.nih.gov [46].

### Statistical Analysis

The mean serum and mucosal antibody levels were compared using a Mann-Whitney non-parametric test. ELISpot SFU of each group were analysed by one-way analysis of variance (ANOVA) with a Fisher's least significant difference multiple-comparison test. A *p* value of <0.05 was considered significant. The SPICE software comparison of distributions was performed using a student's *t* test and a partial permutation test as described [46].

## Results

### Recombinant DNA, MVA and GLA adjuvanted protein elicit substantial vaccine-antigen specific humoral immune responses

Although DNA, MVA and recombinant protein immunogens have been assessed when used sequentially we set out to determine if there was similar or improved immunological outcome when MVA and protein were used in combination to boost DNA priming. In addition, we assessed the impact of GLA-AF, a potent TLR4 agonist. DNA intramuscular vaccinations contained two plasmid constructs (DNA-C), one expressing a codon optimized *gag-pol-nef* transgene derived from the clade C p96ZM651.8 molecular clone and the other a codon optimized *env* transgene from the C/B′ HIV-1 97CN54 isolate. The MVA vector contained a 97CN54 gag-pol-nef fusion transgene and a 97CN54 *env* construct that coded for the gp120 version of Env also from the C/B′ HIV-1 97CN54 isolate. Both transgenes were codon optimized and expressed within one MVA-C construct using back-to-back synthetic early promoters. Vectors were injected into the quadricep muscles and mice where immunized with individual or combined vaccine components as detailed in Table 1.

**Table 1:** Murine vaccination groups.

	Week							
	0	3	6	9	12	15	18	21
A - DNA - MVA -gp140+GLA	DNA	DNA	DNA	-	MVA	MVA	gp140+GLA	gp140+GLA
B - DNA - MVA/gp140+GLA	DNA	DNA	DNA	-	MVA/gp140+GLA	MVA/gp140+GLA		
C - DNA - gp140+GLA	DNA	DNA	DNA	-	gp140+GLA	gp140+GLA		
D - DNA - MVA/gp140	DNA	DNA	DNA	-	MVA/gp140	MVA/gp140		
E - MVA - gp140+GLA	-	-	-	-	MVA	MVA	gp140+GLA	gp140+GLA
F - MVA/gp140+GLA	-	-	-	-	MVA/gp140+GLA	MVA/gp140+GLA		
G - DNA	DNA	DNA	DNA	-	-	-		

Female BALB/c mice received immunizations at three week intervals either sequentially or in combination into different quadricep muscles.

**Figure 1:**
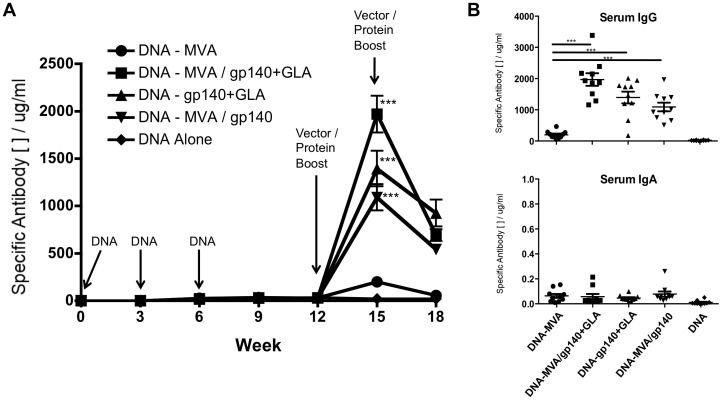
Gp140-Specific Serum IgG responses in mice primed with DNA. (**A**) Animals (*n* = 10) were inoculated 3 times with 100 µg plasmid DNA (IM at week 0, 3 and 6) then boosted with variations of MVA-C (10^7^ PFU) and/or recombinant protein with/without GLA-AF adjuvant (IM inoculation at week 12 and 15). CN54gp140 recombinant protein (10 µg) and GLA-AF adjuvant (20 µg) were admixed and injected into one quadricep muscle. If MVA-C was administered at the same time it was inoculated into the other quadricep muscle. Serum was taken one day prior to each vaccination and the antigen-specific IgG antibody was assessed by an immunoglobulin ELISA with a murine purified IgG standard curve. (**B**) Comparison of the serum IgG or IgA levels in each DNA primed group at peak response (Week 15). Antigen-specific IgG concentrations are shown in µg/ml (+/− SEM). Statistical comparisons were performed using a Mann-Whitney test (***p<0.0001, comparison to DNA – MVA-C group).

Antigen-specific IgG and IgA levels in serum and mucosal samples were determined by semi-quantitative ELISA and the Th1/Th2 indicator isotypes IgG1 and IgG2a were also measured in the serum. We first analysed the immunogenicity of the recombinant MVA-C and the CN54gp140 protein in various combinations with or without adjuvant in those animals that had received plasmid DNA-C priming (Figure 1). While three DNA vaccinations elicited low levels of CN54gp140 antigen-specific serum IgG responses (30 µg/ml at 9 weeks) these were substantially augmented after boosting with recombinant MVA-C vector plus CN54gp140 protein with or without the GLA-AF adjuvant. When compared to animals boosted with recombinant MVA-C alone, the groups that received MVA-C plus recombinant protein had significantly higher antigen-specific serum IgG (***p<0.0001, Mann-Whitney) with the highest levels observed in the group that received MVA-C with GLA-AF adjuvanted protein (mean 1969.7 ug/ml). Furthermore, the peak antigen-specific IgG antibody responses achieved when adjuvanted CN54gp140 protein was administered without the MVA-C vector was also significantly greater than MVA-C boosting alone (***p<0.0001, Mann-Whitney), although lower but not significantly different than animals that received both the MVA-C plus CN54gp10+GLA-AF (DNA – MVA/gp140+GLA vs DNA – gp140+GLA: *p* = 0.063, Mann-Whitney). Therefore, both groups of animals that received recombinant CN54gp140+GLA-AF exhibited the highest levels of serum antigen-specific IgG. Interestingly, animals that received MVA/gp140+GLA inoculations displayed significantly higher responses than those receiving gp140+GLA alone despite the MVA-C alone appearing to be a relatively poor booster of the DNA-C primed immune response. This suggests that the MVA-C component, while not directly augmenting antigen-specific antibody levels, does play a role in enhancing the general immune activation, either by itself or in a combination with the other boost vaccine components. These significant differences observed at peak response three weeks after the first boost inoculation were not maintained when a second boost was given and the antigen-specific IgG serum antibody reduced to similar levels in these three groups after a further three weeks.

**Figure 2:**
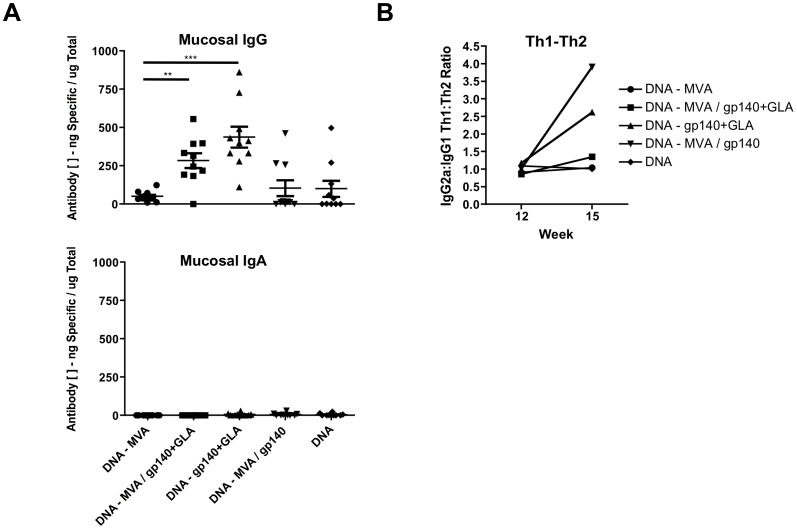
Gp140-Specific Mucosal IgG or IgA responses and serum IgG Th1/Th2 bias in DNA primed mice boosted with various combinations of MVA-C and/or CN54gp140 and GLA-AF. Female BALB/c mice (*n* = 10) inoculated with a total of 100 ug plasmid DNA (50 ug per leg; IM at week 0, 3 and 6) then boosted with variations of 10^7^ PFU MVA-C and/or 10 µg recombinant protein with/without 20 µg GLA-AF adjuvant (IM inoculation at week 12) were assessed at week 15, three weeks after the first boost event. (**A)** Mucosal vaginal wash samples contained moderate levels of vaccine antigen-specific IgG but very low levels of specific IgA (***p<0.0001; **p = 0.0015). (**B**) The effect of the boost inoculation upon the Th1:Th2 bias was assessed with the ratio of the IgG2a∶IgG1 immunoglobulin isotype. Antigen-specific IgG, IgG1, IgG2a or IgA antibody was assessed by an immunoglobulin ELISA with a murine purified IgG, IgG1, IgG2a or IgA standard curve. Antigen-specific Ig concentrations are shown in µg/ml (+/− SEM). Statistical comparisons were performed using a Mann-Whitney test.

We then assessed the isotype profile of the peak serum and mucosal CN54gp140 antigen-specific Ig responses elicited by each vaccination regimen (week 15 after first plasmid DNA-C inoculation; Figure 1). While serum IgG levels (including IgG, IgG1 and IgG2a) were readily detected in all groups only very low levels of specific IgA responses were observed in the serum (Figure 1B). Furthermore, there were only very low levels of vaccine antigen-specific IgA in mucosal wash samples (Figure 2A) but moderate levels of IgG, with greater than 50% of the total mucosally secreted IgG antibody being specific for CN54gp140 in some animals (range 0.3–86% depending on regimen). Interestingly the highest level of mucosally sourced IgG was not detected in the DNA – MVA/gp140+GLA group which had the highest serum antigen-specific IgG response, but rather in the group that had received adjuvanted protein only without MVA. To further characterize the vaccine-elicited humoral response we assessed the potential skewing of the immune response using the IgG2a∶IgG1 ratio as a surrogate for Th1:Th2 bias (Figure 2B). We measured the IgG1 and IgG2a immunoglobulin isotypes at week 12, when the animals had received the three DNA-C priming vaccinations, and then again at week 15, three weeks after they had been boosted. Animals that were boosted with the MVA-C vector alone showed no alteration to the bias of the immune response, however all groups that received a recombinant protein boost demonstrated some skewing towards Th2, a known consequence of DNA prime/protein boosting in BALB/c mice [47]. However, the presence of the TLR4 agonist GLA-AF prevented or reduced this strong bias as previously described [48], [49].

### Anamnestic humoral responses to recombinant CN54gp140 protein or MVA after plasmid DNA priming

**Figure 3:**
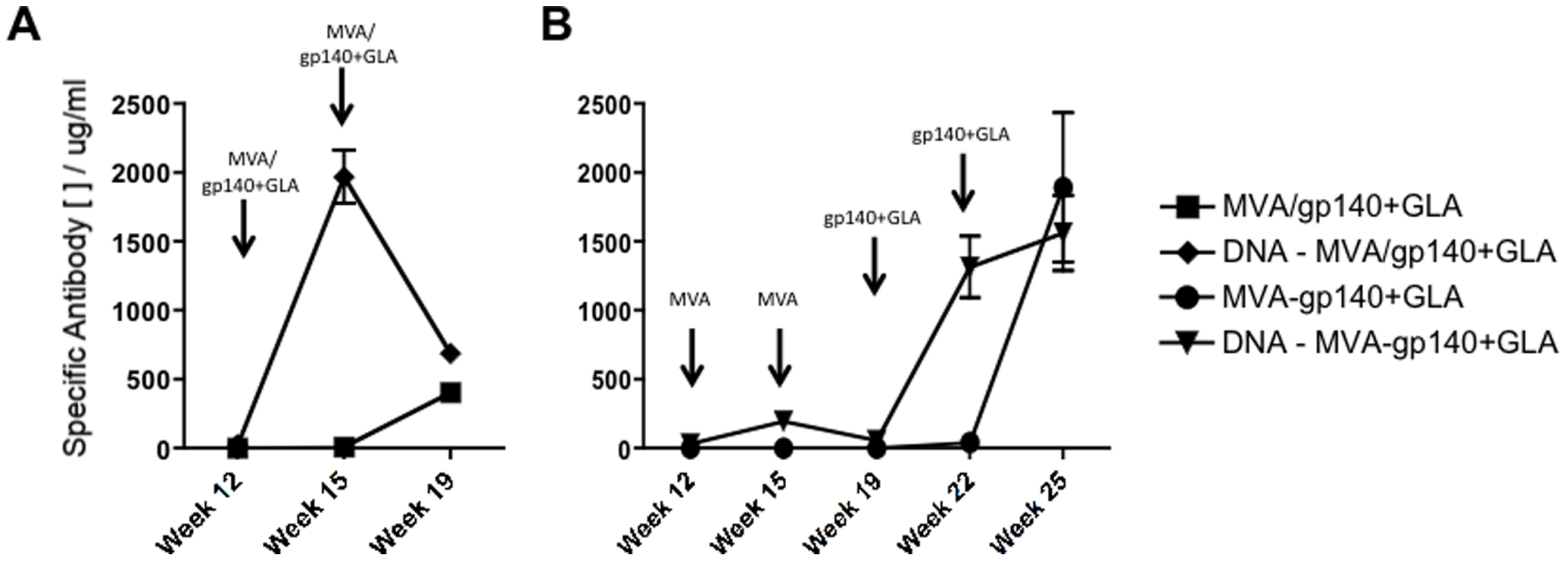
Anamnestic humoral responses after plasmid DNA vaccination. (**A**) Mice that had been previously inoculated three times with plasmid DNA expressing the CN54gp140 *Env* transgene elicited a memory B cell population that differentiated into antigen-specific antibody secreting cells after a single MVA/gp140+GLA vaccination. (**B**) MVA alone was also able to re-stimulate this memory population of B cells and this was again further boosted by subsequent inoculations of the recombinant adjuvanted antigen CN54gp140. Antigen-specific IgG antibody was assessed by an immunoglobulin ELISA and concentrations are shown in µg/ml (+/− SEM).

We assessed the contribution of the plasmid DNA within our vaccine regimen by comparing the vaccine-generated immune responses of groups of animals that did not receive DNA-C priming inoculations with those that received three DNA-C prime vaccinations (Table 1). We first compared those animals that received the MVA-C vector together with the adjuvanted CN54gp140 protein and observed a clear anamnestic response after the boost inoculation at week 12, demonstrating that the DNA-C priming inoculation did indeed elicit a population of antigen-responsive immune cells (Figure 3A). Those animals that did not receive the plasmid DNA-C prime vaccination did not respond until after the second administration of MVA/gp140+GLA and even then the level of the peak response in this cohort was less than 25% of the peak value measured in the DNA primed animals.

We next looked at those groups of animals where the boost inoculations were administered separately at three-week intervals rather than given together. We noted that the MVA-C vector was able to boost the antigen-specific immune response in DNA-C experienced animals, though the second MVA-C inoculation appeared to have a detrimental effect, possibly due to an insufficient gap between the boost vaccinations and/or potential response modulation by the many immune control factors expressed by attenuated MVA [50] (Figure 3B). Interestingly, the MVA-C was a poor primer of immunity and the group of animals that had not received any DNA-C priming immunizations (which were effectively naïve) responded weakly to inoculation with MVA-C vector alone, while a reasonable level of antigen-specific immunity was achieved in this group after the second adjuvanted protein inoculation. At the end of the series of vaccinations however, both regimens (+/− DNA-C priming) elicited similar levels of antigen-specific serum immunoglobulin.

### T cell responses to vector-derived antigens were altered in the presence of the GLA-AF adjuvant in mice

**Figure 4:**
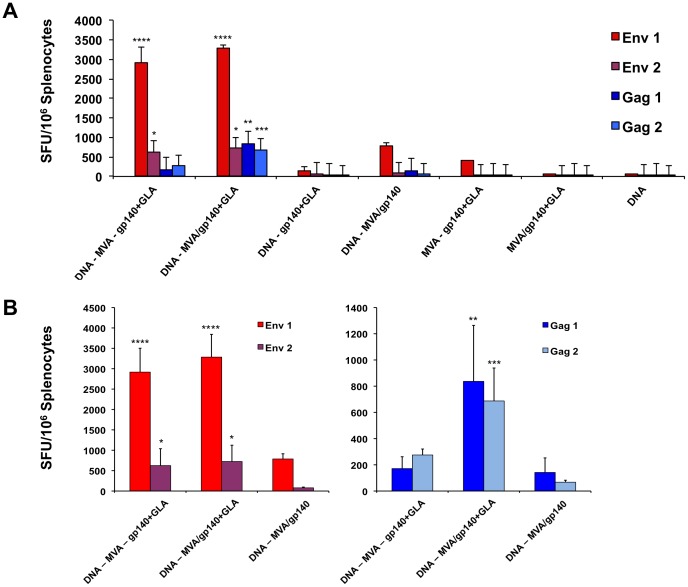
Splenocyte IFN-γ ELISpot responses to specific vaccine antigen derived peptide pools. Cells isolated from mouse spleens at termination were stimulated with peptide pools (15 mers overlapping by 11 aa) that covered the entire coding sequence of the Gag and Env protein (two pools for each peptide) that were expressed from the transgenes present in the two plasmid DNAs and the MVA-C vector. (**A**) Specific peptide pool responses of splenocytes from all study groups. (**B**) Comparison of splenocyte IFN-γ ELISpot responses to the Env and the Gag peptide pools in animals that had been DNA primed then inoculated with either MVA-C and CN54gp140+GLA sequentially or with the MVA and protein or with all three boost vaccine components concurrently. Responses from each group of animals are shown as group mean spot forming units (SFU)/million splenocytes +/− SEM. Groups were statistically compared using a one-way Anova (*p<0.05; **p<0.01; ***p<0.005; **** *p*<0.001).

Subsequently we evaluated the cellular immune response in the systemic compartment of these various vaccine regimen groups (Table 1). An IFN-γ ELISpot assay of splenocytes from each animal demonstrated that both the DNA – MVA – gp140+GLA and the DNA – MVA/gp140+GLA groups, where the recombinant adjuvant was given sequentially or in a regime shortening combination, developed CN54gp140 envelope-specific T cell responses that were dramatically higher than the other vaccine groups (Figure 4A) suggesting that the presence of all vaccine components was required for optimal T cell immune responses. The response to peptides contained within the Env1 pool (****p<0.001; ANOVA) were significantly greater than the Env2 Pool suggesting the presence of one or more immunodominant T cell epitopes within the first half of the CN54 envelope protein. Splenocyte responses to peptides in the Env2 pool were also significantly higher than those to the Gag peptide pools in the two groups that had received all the vaccine components (*p<0.05; ANOVA). Interestingly however, the only vaccination group to demonstrate significantly higher Gag-specific T cell responses than the DNA vaccine alone group was the regime shortening DNA – MVA/gp140+GLA cohort where the GLA adjuvant was given concurrently (though in a separate leg) with the MVA-C vector (Gag 1 - **p<0.01; Gag 2 – ***p<0.005, ANOVA). The gag antigen was only expressed in the DNA-C plasmid and the MVA-C vector, strongly suggesting that the GLA-AF TLR4 adjuvant, in addition to augmenting the humoral immune response to the recombinant CN54gp140 protein was also able to act systemically and enhance the cellular immune response to the MVA-C vector expressed transgenes (Figure 4B).

**Figure 5:**
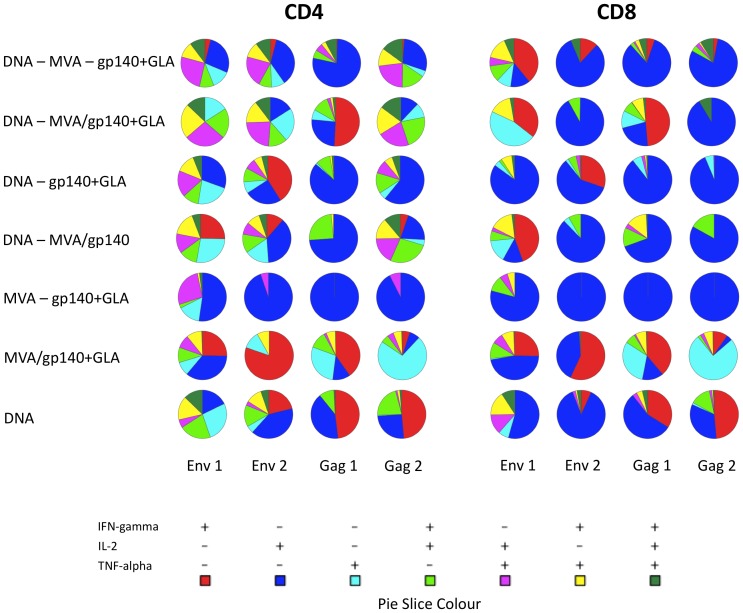
Polyfunctional cytokine responses of peptide pool stimulated splenocytes. Cells from each animal were stimulated with each different peptide pool for 6°C in the presence of a golgi secretion inhibitor. CD4+ or CD8+ T cell populations were gated and single, double or triple cytokine expressing cell percentage enumerated. The percentage cytokine expression of each animal was averaged within the group and is expressed as a pie-chart of relative proportions of the total cytokine expressing cell population. Pie slice colour key indicates the particular cytokine(s) expressed.

We then used intracellular cytokine staining (ICS) to assess the polyfunctionality or cytokine response profile of CD4 and CD8 T cells from each vaccination group. From these analyses it was clear that the CD4 splenocyte T cells displayed a greater degree of polyfunctionality than CD8 T cells, with very low percentages of CD8+ cells expressing all three cytokines, IFN-γ, TNF-α and IL-2 (Figure 5 and Table S1). Animals primed with plasmid DNA-C contained moderate populations of cells expressing all three cytokines whereas those animals that did not receive DNA-C priming exhibited no triple-expressors, strongly suggesting a role for DNA-C priming in broadening the cellular immune response. Of particular note was the observation that vaccination groups that received all vaccine components demonstrated the greatest diversity of cytokine expression and contained the largest populations of double- and triple-cytokine expressing CD4 T cells. Both the sequential DNA – MVA – gp140+GLA and the regime shortened DNA – MVA/gp140+GLA groups contained highly polyfunctional cytokine expressing populations that responded to the Env1, Env2 and the Gag2 peptide pools, suggesting that these regimens were able to elicit a more divergent vaccine-antigen specific population of T cells.

### The vaccine components elicited high antigen-specific serum IgG in rabbits

**Figure 6:**
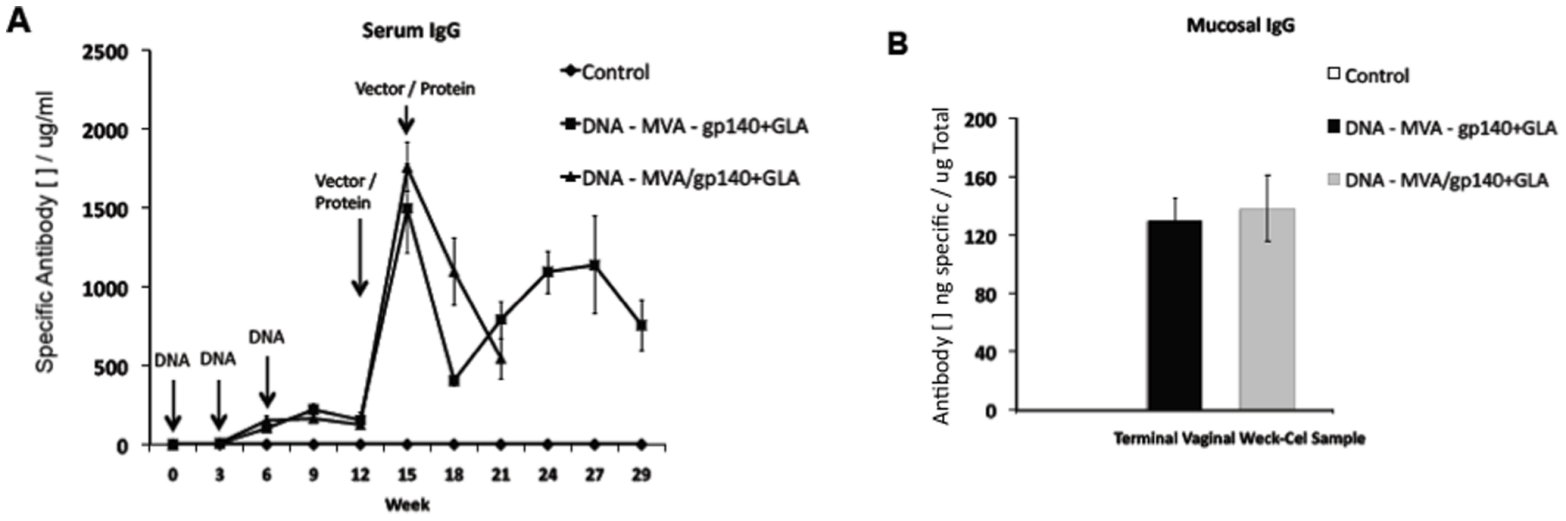
Vaccine elicited CN54gp140-specific serum and mucosal IgG responses in rabbits. Male and female NZW rabbits were primed with three DNA inoculations (8 mg total; 4 mg each leg) into their quadricep muscles then boosted with either MVA – gp140+GLA given sequentially or MVA/gp140+GLA administered concurrently in separate leg muscles (MVA-C at 1.3×10^8^ PFU in 500 µl; CN54gp140 at 100 µg in 400 µl; GLA-AF at 5 µg in 400 µl – co-formulated with CN54gp140 recombinant protein). The antigen-specific serum IgG response over the course of the vaccine regimen (**A**) and the mucosal IgG response at termination (**B**). Antigen-specific rabbit IgG antibody was assessed by an immunoglobulin ELISA with a rabbit purified IgG standard curve. Antigen-specific IgG concentrations are shown in µg/ml (+/− SEM).

The sequential and shortened vaccine regimens were then assessed in a second species to determine whether the very high levels of serum antigen-specific antibody were attainable in animals other than mice. We injected male and female rabbits with 4 mg of each of the naked plasmid DNA-Cs (CN54 *Env* in the left hind leg quadriceps and ZM96 *Gag-pol-nef* in the right hind quadriceps) followed by 1.3×10^8^ PFU MVA-C either alone or along with 100 µg CN54gp140+5 µg GLA-AF according to the vaccination schedule in Table 2, as part of a toxicological and immunogenicity analysis in preparation for translation into a clinical trial. The CN54gp140-specific serum IgG responses demonstrated that these vaccine regimens elicited comparably high humoral responses to those seen in mice, with no statistically significant difference between the sequential and the shortened regimes (Figure 6A). Furthermore, and in broad accordance with the initial murine studies, the level of mucosally sourced antigen-specific IgG was 10–20% of the total immunoglobulin present in the rabbit genital tract (Figure 6B). Mucosal IgA was analysed but none was detected in vaginal samples from these animals (data not shown).

**Table 2:** Rabbit vaccination groups.

	Week							
	0	3	6	9	12	15	18	21
1 - Vehicle Control	PBS	PBS	PBS	-	Dil Buffer	Dil Buffer	Dil Buffer	Dil Buffer
2 - DNA - MVA - gp140+GLA	DNA	DNA	DNA	-	MVA	MVA	gp140+GLA	gp140+GLA
3 - DNA - MVA/gp140+GLA	DNA	DNA	DNA	-	MVA/gp140+GLA	MVA/gp140+GLA		

Male and female NZW rabbits received immunizations at three week intervals either sequentially or in combination into different quadricep muscles. The reduced quantity of GLA-AF when compared with the murine experiments more closely models the amount that will be administered in humans, a refinement based on our repeat experiments where we found that immune stimulation elicited by 5 ug GLA-AF was equivalent to 20 ug GLA-AF. The regime-shortened group DNA-MVA/gp140+GLA animals were euthanized at week 21, while the sequential immunisation and control animals were euthanized at week 29, to fulfil the requirement for toxological analysis.

## Discussion

We set out to determine the relative immunogenicity of the different components of a DNA-MVA-Protein (+/− adjuvant) vaccine regimen in order to design an optimal immunization regime for a phase I human clinical trial. We wanted to assess whether a regime shortening approach was equivalent or better than sequential vaccinations in generating vaccine-antigen specific immunity. We performed a number of regimen permutations, each designed to investigate the contribution of individual components of a heterologous vector but HIV Clade C antigen-matched strategy, and assessed the vaccine-elicited responses in both cellular and humoral compartments. Quantitatively, we measured levels of serum and mucosal antibody and the degree of T cell reactivity and qualitatively we assessed antibody isotype skewing and the polyfunctionality of the antigen-specific cellular response.

In agreement with previous publications, naked plasmid DNA-C vaccinations elicited moderate levels of antigen-specific IgG (≥30 ug/ml) [51]. In addition, DNA-C priming was able to effectively generate and establish a population of memory B cells that could be effectively expanded with either a recombinant vector boost inoculation that expressed the protein from encoded transgenes or administration of recombinant protein itself, an effect that was confirmed in a subsequent rabbit immunogenicity experiment. The boost components of the vaccine regimen were also able to elicit antigen-specific humoral responses when given without any DNA-C prime vaccination, though the MVA-C vector generated low-level responses, likely consistent with a better ability to promote cellular immunity. As we have previously observed, intramuscular injection of recombinant proteins elicit little antigen-specific IgA, with only very low levels detected in the serum and none in mucosal secretions [52].

An analysis of splenic T cell responses demonstrated that the DNA-C priming contributed very significantly to the overall magnitude of the cellular immune response. Animals that had not received any DNA-C prime inoculations had very low levels of T cell reactivity, barely registering above background. In agreement with published work performed in both mice and humans that demonstrated dominant T cell responses to the V1/V2 segment and the V3 loop of the HIV envelope protein we found high splenocyte T cell reactivity to the Env 1 peptide set that spans these regions of the CN54gp140 vaccine antigen [53]–[55]. We also carried out an analysis of the polyfunctionality, or functional diversity of the cellular immune response. Those animals that had been primed with DNA-C all demonstrated a greater degree of polyfunctional CD4 and CD8 T cell responses at the end of their vaccination regimen. In contrast, the animals that received MVA-C and HIV CN54gp140 protein without DNA-C priming, whether within a shortened or sequential regimen had T cells that exhibited a limited ability to secrete more than one cytokine, with a majority of the cells analysed being monocytokine expressors. Conversely, the groups that had an additional prior DNA-C priming vaccination contained majority populations of multicytokine expressors, particularly in the CD4 T cell compartment.

Interestingly, administration of the GLA-AF adjuvant+CN54gp140 at the same time as the MVA-C vector, although injected into different hind leg quadricep muscles, was able to significantly augment the immune response to transgene products only expressed by the vector and not present in the GLA-AF co-formulated recombinant protein. As the components were administered into different legs of the animals this observation strongly suggests that the GLA-AF has systemic adjuvant effects, heightening the immune response to the transgenes expressed by the MVA-C vector. An additional effect of the GLA-AF adjuvant was a modification of the degree of Th1:Th2 skewing of the humoral response. In groups that received DNA or DNA – MVA inoculations we observed no bias in the immune response but those groups that had received any recombinant protein vaccinations as part of the regimen demonstrated clear skewing toward a Th2 biased response. We were able to show with the addition of the TLR4 agonist that this strong Th2 bias was prevented, demonstrating the potential utility of GLA-AF to manipulate or ‘tailor’ immune responses.

Taken together these results strongly indicate that the DNA-C inoculations enhance the breadth of the immune response and also that this diversity is not swamped by subsequent vaccination with adjuvanted protein, as the T cell cytokine analysis was performed at the end of the vaccination schedule. We have shown that these vaccine components are immunogenic in both mice and rabbits and are able to elicit high levels of humoral responses and large numbers of systemic antigen-specific T cells, particularly to the envelope protein. We have further demonstrated that the concurrent administration of a vectored vaccine with a GLA-AF adjuvanted recombinant protein has significant and unexpected advantages, expanding responses to the vector expressed antigens and promoting the elicitation of a polyfunctional cellular response. Moreover, there are clear benefits of a shortened vaccine regimen that is equivalent or immunologically preferential to a sequential multi-visit dosing schedule, through increased compliance and lower costs.

## Supporting Information

Table S1 **Percentage positive of gated and live CD4 and CD8 T cells that secreted cytokine in response to peptide pools Env 1, Env 2, Gag 1 or Gag 2.**
(TIF)
