# Supplementary figures and images for: Glucopyranosyl Lipid A Adjuvant Significantly Enhances HIV Specific T and B Cell Responses Elicited by a DNA-MVA-Protein Vaccine Regimen
Source: PLoS One. 2014 Jan 23;9(1):e84707. doi: 10.1371/journal.pone.0084707 (PMC3900398; doi:10.1371/journal.pone.0084707)

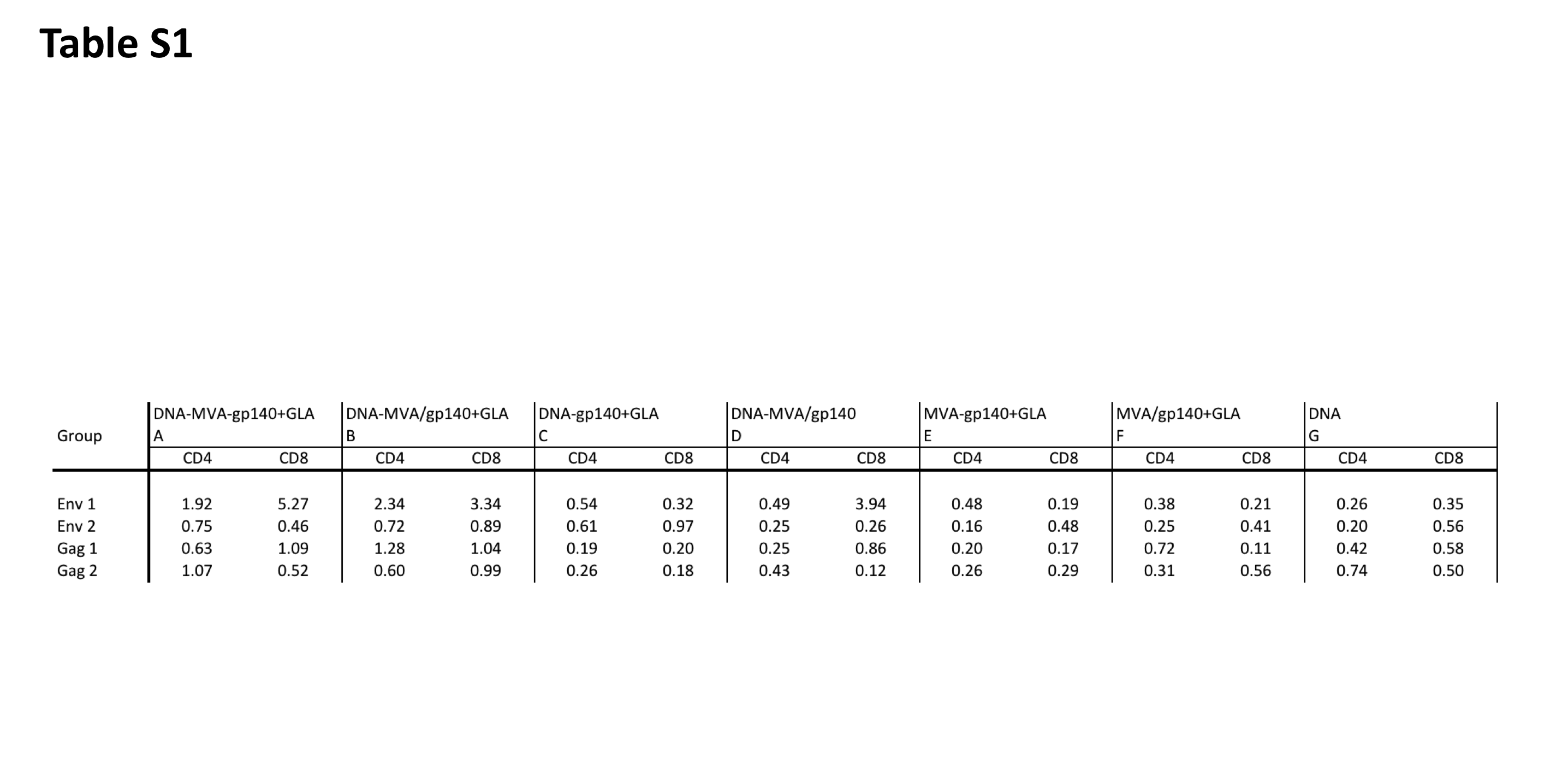

Supplement: Table S1 — Percentage positive of gated and live CD4 and CD8 T cells that secreted cytokine in response to peptide pools Env 1, Env 2, Gag 1 or Gag 2. (TIF) [file pone.0084707.s001.tif]
